# Impact of enhanced recovery program on clinical outcomes after elective colorectal surgery in a rural hospital. A single center experience^☆^

**DOI:** 10.1016/j.heliyon.2024.e33989

**Published:** 2024-07-03

**Authors:** Antonio Pesce, Mattia Portinari, Nicolò Fabbri, Valeria Sciascia, Lisa Uccellatori, Michela Vozza, Erminio Righini, Carlo V. Feo

**Affiliations:** a Unit of General Surgery, Department of Surgery, Azienda Unità Sanitaria Locale of Ferrara, University of Ferrara, Ferrara, Italy; b Unit of Surgery 2, Department of Surgery, S. Anna University Hospital of Ferrara, Ferrara, Italy; c Unit of Anesthesia and Intensive Care, Department of Emergency, Azienda Unità Sanitaria Locale of Ferrara, Ferrara, Italy

**Keywords:** Enhanced recovery program, Colorectal surgery, Clinical outcomes, Rural hospital, Length of stay

## Abstract

**Background:**

The main purpose was to determine the impact on postoperative outcomes of a standardized enhanced recovery program (ERP) for elective colorectal surgery in a rural hospital.

**Methods:**

A prospective series of patients (N = 80) undergoing elective colorectal resection completing a standardized ERP protocol in 2018–2020 (ERP group) was compared to patients (N = 80) operated at the same rural hospital in 2013–2015 (pre-ERP group), before the implementation of the program. The exclusion criteria for both groups were: ASA score IV, TNM stage IV, inflammatory bowel disease, emergency surgery, and rectal cancer. The primary outcome was hospital length of stay (LoS) which was used as an estimate of functional recovery. Secondary outcomes included 30-day readmission and mortality rates as well as associated factors with both postoperative complications and prolonged hospital LoS.

**Results:**

Baseline characteristics were comparable in both groups. The median adherence to ERP protocol elements was 68 % versus 12 % in the retrospective control group. The median hospital LoS in the ERP-group was significantly lower than in the pre-ERP group (5 *vs*. 10 days) with no increase in 30-day readmission and mortality rates. The Body Mass Index ≥30 and the traditional peri-operative protocol were the associated factors to postoperative complications, while following a traditional peri-operative protocol was the only factor associated with a prolonged hospital LoS (p < 0.0001).

**Conclusions:**

Although limited hospital resources are perceived as a barrier to ERP implementation, the current experience demonstrates how adopting an ERP program in a rural area is feasible and effective, despite it requires greater effort.

## 1 Introduction

The Enhanced Recovery Program (ERP) is a scientific evidence-based peri-operative care approach centered on a multidisciplinary team aiming to improve postoperative outcomes and to reduce recovery time in surgical patients, by attenuating the peri-operative metabolic response and organ dysfunction [1,2].

The ERP protocols have primarily been developed and used in urban and academic centers in Europe and North America. Until today, however, there are very few data concerning the application of ERP in elective colorectal surgery in rural and community-based hospitals, serving wide and remote rural areas, where medical resources may be limited [[3], [4], [5], [6]]. People living in a rural environment present many differences from those living in urban areas, in terms of social (e.g., degree of education, health literacy, transportation) and health (e.g., access to medical care, co-morbidities, post-discharge facilities) factors. Furthermore, rural areas are generally larger and less densely populated than urban ones and this implies potential difficulties both for hospital discharge and for patient care [7,8]. Another important aspect is the case-volume, which may be lower in rural hospitals, and the higher medical and nursing staff turnover and shortage.

As scientific evidence concerning the effectiveness of ERP in rural contexts is limited, the primary objective of this study was to determine the impact on postoperative outcomes of a standardized ERP for elective colorectal surgery in a rural, teaching community hospital using the length of stay (LoS) as a surrogate of functional recovery. The secondary outcomes were to evaluate the 30-day readmission and mortality rates as well as to identify the associated factors with both postoperative complications and prolonged hospital LoS.

## 2 Methods

### 2.1 Study design, setting, participants

A prospective series of consecutive patients (N = 80) undergoing elective colorectal resection completing a standardized ERP protocol from November 2018 to July 2020 (ERP group) was compared to patients (N = 80) operated at the same hospital in Northern-East of Italy from April 2013 to December 2015 (Pre-ERP group), before the implementation of the protocol. The year 2016 was excluded due the retirement of the colorectal surgeon operating in the period 2013–2015, while throughout the year 2017 the ERP protocol was implemented. We decided to exclude patients in 2016 due to a lower number of colorectal resections and the bias of multiple first-operator surgeons with heterogeneous clinical practices.

One fully trained surgeon in colorectal surgery did all open cases in the Pre-ERP group and another minimally invasive surgery-trained colorectal surgeon did all the cases in the ERP group. Moreover, the ERP group surgeon (C.V.F.) had also a consolidated experience implementing ERPs [[9], [10], [11]].

Eligible criteria were: age ≥18 years-old, elective colorectal resection, American Society of Anesthesiologists (ASA) score I-III. The exclusion criteria for both groups were: ASA score IV, TNM stage IV, inflammatory bowel disease, emergency surgery, and rectal cancer.

### 2.2 Variables and data sources

Data were recorded prospectively in the ERP group, while they were retrospectively extracted from medical record documentation in the Pre-ERP group by two separate investigators (L.S., V.S.) who were blinded to the study protocol.

All complications were recorded until 30 days after surgery, as well as mortality and hospital readmission.

Patients in both groups were discharged after full recovery from the surgical operation. From postoperative day 3 (targeted discharge day), the patients were evaluated for early discharge. The adopted parameters for patient recovery were as follows: 1) Complete oral feeding recovery, without any restriction; 2) Complete gastrointestinal recovery, defined as the time taken for patients to tolerate solid food and to pass stool; 3) Complete dynamic pain control with oral analgesics (i.e., Numerical Rate Scale – NRS ≤3); 4) Return to complete mobilization after surgery; 5) No local or systemic sign of infection.

The Clavien–Dindo grading system [12] was used to classify each patient's most severe encountered complication: no complication (grade 0), minor complication (grades I–II) or major complication (grades III–V).

The applied items included in the protocol, derived from the *fast-track* one proposed by Kehlet and Wilmore in the mid-1990s,^2^ are listed in Table 1. All ERP items were listed in a specific checklist and were recorded in all postoperative days until patient discharge. The medical records of Pre-ERP patients were reviewed to evaluate adherence to the elements comprised in the ERP protocol to determine how much the clinical practice had been modified by its implementation.

**Table 1:** Key elements of the Enhanced Recovery Program (ERP) protocol.

Variables	ERP Group (N=80)	Non-ERP Group (N=80)	p-value
Pre-admission counselling [N(%)]	79 (98,7)	0 (0)	<0.0001
Information booklet [N(%)]	79 (98,7)	0 (0)	<0.0001
No mechanical bowel preparation [N(%)]	80 (100)	28 (35)	<0.0001
No pre-operative fasting [N(%)]	80 (100)	0 (0)	<0.0001
Pre-operative oral carbohydrate loading [N(%)]	78 (97,5)	0 (0)	<0.0001
No premedication [N(%)]	71 (88,7)	80 (100)	0.01
Mid-thoracic epidural anesthesia [N(%)]	34 (42,5)	22 (27,5)	0.06
Preoperative TAP-block [N(%)]	44 (55)	0 (0)	<0.0001
Short-acting anesthetic agent [N(%)]	80 (100)	0 (0)	<0.0001
Avoidance of intraoperative fluids overload (<5 ml/kg/h)	1 (1,25)	0 (0)	0.99
Intraoperative maintenance of normothermia [N(%)]	80 (100)	80 (100)	0.99
Prevention of nausea and vomiting [N(%)]	47 (58,7)	3 (3,75)	<0.0001
Minimally invasive surgery [N(%)]	76 (95)	0 (0)	<0.0001
No abdominal drains [N(%)]	59 (73,7)	0 (0)	<0.0001
No nasogastric tube [N(%)]	51 (63,7)	7 (8,7)	<0.0001
Early mobilization (day ≤2) [N(%)]	19 (23,7)	1 (1,25)	<0.0001
Post-operative breathing exercises [N(%)]	80 (100)	0 (0)	<0.0001
Mid-thoracic epidural analgesia [N(%)]	34 (42,5)	22 (27,5)	0.06
Non-opiate oral analgesics/NSAIDs [N(%)]	66 (82,5)	27 (33,7)	<0.0001
Stimulation of gut motility [N(%)]	36 (45)	1 (27,5)	<0.0001
Early removal of bladder catheter (day ≤2) [N(%)]	47 (58,7)	4 (5)	<0.0001
Early oral nutrition (day ≤1) [N(%)]	55 (68,7)	0 (0)	<0.0001

TAP: transverse abdominis plane; NSAIDs: non-steroidal anti-inflammatory drugs; Pre-operative fasting: from midnight; Early oral nutrition: from the same day of surgery.

### 2.3 Compliance with ethical standards

The study was carried out in accordance with the International Ethical Guidelines and Declaration of Helsinki. All patients signed a written informed consent before surgery. The study protocol (ID: 354/2019/Oss/AUSLFe) was approved by the local Ethical Committee (*Comitato Etico Area Vasta Emilia Centro*– CE-AVEC).

### 2.4 Statistical analysis

Clinical parameters were expressed as median [interquartile range (IQR) 25–75)] according to distribution assessed by Shapiro–Wilk test. Categorical data were presented as numbers. Clinical and pathological variables were analyzed with chi-square, and Mann–Whitney tests as appropriate. The Kaplan–Meier test method and Log-Rank test were used to compare the duration of surgical operation, time to complete functional recovery, and hospital LoS between the two groups. A logistic regression analysis was performed to evaluate the associated factors with postoperative complications, while the independent factors of prolonged hospital LoS were determined by using a Cox regression analysis. Surgical approach (laparoscopy versus open), intraoperative fluid infusions and duration of surgery were the potential confounders used in the logistic regression analysis and in the Cox regression. Of note, hazard ratios (HRs) < 1 correspond to an association of the factor with prolonged hospital LoS, while HRs >1 correspond to earlier discharge. A p-value <0.05 was considered statistically significant. All collected data were included in an electronic study database and analyzed using the SPSS software (IBM SPSS Statistics for Windows, version 20.0). This report complies with the reporting standards established by STrengthening the Reporting of OBservational studies in Epidemiology (STROBE) guidelines for reporting observational studies.

## 3 Results

A total of 160 patients undergoing elective colonic resections at our institution were included in this analysis. The investigation group (ERP group) comprised 80 patients operated on *after* the implementation of the colorectal ERP, in 2017, while the control group (Pre-ERP group) included 80 patients operated on *before* starting the ERP.

**Fig. 1:**
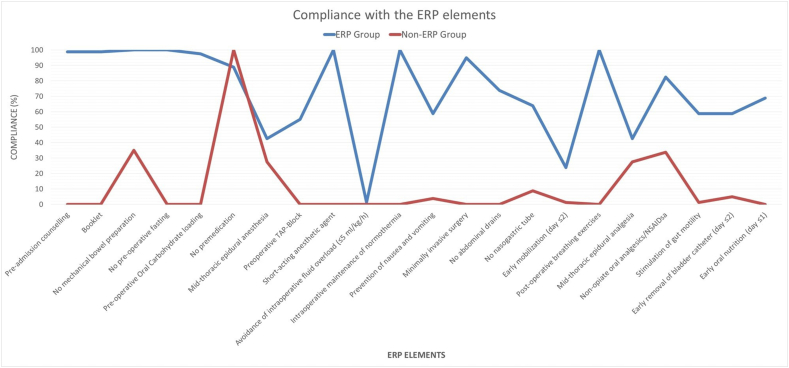
Compliance with ERP protocol.

The median adherence to ERP protocol was 68 % as opposed to 12 % of the retrospective control group. Avoidance of intra-operative fluid overload and delayed early mobilization of patients after surgery were the main elements of lower compliance in the ERP group, as shown in Fig. 1.

Demographic and clinical data are reported in Table 2. Baseline characteristics were comparable in both groups, except for chronic kidney insufficiency, which was significantly more frequent in the ERP group. Laparoscopic approach was performed in 95 % of patients in the ERP group versus 0 % in pre-ERP group (p-value <0.0001). Among intra-operative variables, prophylactic naso-gastric tube and abdominal drains placement were lower in ERP group compared to Pre-ERP group (p-value <0.0001); the rate of intra-operative fluids infusion was about 5 ml/kg/h lower in ERP group (p-value <0.0001).

**Table 2:** Demographic and baseline characteristics.

Variables	ERP Group (N=80)	Non-ERP Group (N=80)	p-value
**Gender**[N(%)]			0.42
Male	43 (53,7)	49 (61,2)	
Female	37 (46,3)	31 (38,8)	
**Age** (years)[N(%)]			0.62
median (IQR 25-75)	77 (69-83)	78 (69-83)	
< 65	15 (18,7)	13 (16,2)	
65-74	21 (26,2)	17 (21,2)	
≥ 75	44 (55,1)	50 (62,6)	
**BMI** (Kg/m^2^)[N(%)]			0.45
< 25	30 (24)	30 (24)	
25-29.9	39 (31,2)	33 (26,4)	
≥ 30	11 (13,7)	17 (13,6)	
**ASA score**[N(%)]			0.39
I	2 (2,5)	3 (3,7)	
II	33 (41,2)	41 (51,2)	
III	45 (56,3)	36 (45,1)	
**History of Diabetes**[N(%)]	12 (15)	16 (20)	0.53
**Hypertension**[N(%)]	54 (67,5)	48 (72,5)	0.41
**Asthma**[N(%)]	2 (2,5)	0 (0)	0.49
**COPD**[N(%)]	8 (10)	6 (7,5)	0.78
**Valvular heart disease**[N(%)]	6 (7,5)	1 (1,2)	0.11
**Ischemic heart disease**[N(%)]	9 (11,2)	7 (8,7)	0.79
**Atrial fibrillation**[N(%)]	8 (10)	14 (17,5)	0.25
**Hypercholesterolemia**[N(%)]	19 (23,7)	11 (13,7)	0.15
**Chronic kidney disease** [N(%)]	11(13,7)	2 (2,5)	0.01
**Depressive disorder**[N(%)]	7 (8,7)	6 (7,5)	0.99
**MUST score**			0.64
0	54	72	
1	15	4	
2	9	4	
3	1	0	
4	1	0	
**Pre-operative haemoglobin levels** [median (IQR 25-75)]	12.0 (10.9-13.2)	11.2 (10.0-13.4)	0.13

BMI, body mass index; ASA, American Society of Anaesthesiologists; COPD: Chronic obstructive pulmonary disease; MUST: Malnutrition Universal Screening Tool.

All intra-operative variables are shown in Table 3.

**Table 3:** Intra-operative variables.

Variables	ERP Group (N=80)	Non-ERP Group (N=80)	p-value
**Disease**[N(%)]			0.99
Malignancy	68 (85)	69 (86)	
Benign tumors/Diverticular disease	12 (15)	11 (14)	
**Type of operation**[N(%)]			0.70
Right colectomy	50 (62)	52 (65)	
Left colectomy	5 (6)	3 (4)	
Transverse colectomy	3 (4)	4 (5)	
Segmental colonic resection	6 (7)	8 (10)	
Sigmoidectomy	15 (19)	10 (12)	
Recto-sigmoid resection	1 (1,2)	3 (4)	
**Preoperative TAP-Block**[N(%)]	44 (55)	0 (0)	<0.0001
**Formation of new stoma**[N(%)]	4 (5)	1 (1,2)	0.36
**Surgical approach**[N(%)]			<0.0001
Laparotomy	0 (0)	80 (100)	
Laparoscopy	76 (95)	0 (0)	
Laparoscopy with conversion	4 (5)	0 (0)	
**Length of procedure** (min)^a^	170 (153-200)	80 (65-90)	<0.0001
**Intraoperative intravenous fluids** (ml/kg/h)^a^	11.5 (8.5-14.6)	16.1 (12.9-22.2)	<0.0001
**Intraoperative RBC transfusion**[N(%)]	1 (1,2)	5 (6)	0.21

a Median (IQR25-75); TAP: Tranverse Abdominis Plane; RBC: Red Blood Cells.

All the measured post-operative outcomes are reported in Table 4. Among them, the complete gastrointestinal recovery was achieved earlier in the ERP group, as well as early mobilization and pain control by oral analgesics (p-value <0.0001). The median hospital LoS in ERP-group was 5 days (4–7 days) versus 10 days (9–14 days) in the pre-ERP group. ERP protocol determined a reduction of 31 % in post-operative complications, mostly grade I and grade II. No significant difference in mortality and 30-days re-admission rates was found between the two groups.

**Table 4:** Measured postoperative variables and outcomes.

Variables	ERP Group (N=80)	Non-ERP Group (N=80)	p-value
**Positioning of** [N(%)]			
Central venous catheter	9 (11)	19 (24)	0.06
Epidural catheter	34 (42	22 (27)	0.06
Prophylactic nasogastric tube (NGT)	29 (36)	73 (91)	<0.0001
Abdominal drain	21 (26)	80 (100)	<0.0001
**Day of removal of** [median (IQR 25-75)]			
Epidural catheter	3 (2-3)	2 (1-3)	0.20
NGT	1 (1-2)	3 (2-4)	0.01
Abdominal drain	3 (3-5)	7 (6-8)	<0.0001
Foley catheter	2 (2-3)	7 (5-9)	<0.0001
**Post-operative RBC transfusion**[N(%)]	5 (6)	21 (26)	0.01
**Vomiting ≤ 24 h** [N(%)]	12 (15)	9 (11)	0.49
**Vomiting > 24 h**[N(%)]	7 (9)	24 (30)	0.01
**Reactive NGT**[N(%)]	11 (14)	12 (15)	0.99
**Resumption of intravenous fluids**[N(%)]	4 (5)	2 (2,5)	0.44
**Postoperative intravenous opioids** [N(%)]	14 (17)	53 (66)	<0.0001
**ICU admission**[N(%)]	38 (47)	16 (20)	<0.0001
**Median ICU length of stay** (days) [median (IQR 25-75)]	1 (1-1)	2 (1-4)	0.001
**Stimulation of gut motility by chewing-gum**[N(%)]	36 (45)	1 (1,2)	<0.0001
**Oral liquid intake** (day) [median (IQR 25-75)]	1 (1-2)	5 (4-6)	<0.0001
**Oral solid intake** (day) [median (IQR 25-75)]	3 (2-4)	7 (6-8)	<0.0001
**Time to intestinal activity** (day) [median (IQR 25-75)]	2 (1-2)	3 (2-4)	<0.0001
**Time to bowel movements** (day) [median (IQR 25-75)]	3 (2-4)	5 (4-6)	<0.0001
**Time to optimal pain control with oral analgesics** (day) [median (IQR 25-75)]	3 (3-4)	4 (3-7)	<0.0001
**Early mobilization** (day) [median (IQR 25-75)]	3 (2-5)	7 (5-9)	<0.0001
**Fit for discharge** (day)	5 (4-6)	9 (8-12)	<0.0001
**Hospital lenght of stay** (days) [median (IQR 25-75)]	5 (4-7)	10 (9-14)	<0.0001
**Postoperative complications** (Clavien-Dindo) [N(%)]			0.08
Grade I	8 (10)	15 (19)	
Grade II	26 (32)	33 (41)	
Grade IIIa	2 (2)	1 (1,2)	
Grade IIIb	1 (1,2)	4 (5)	
Grade IVa	0 (0)	2 (2)	
Grade IVb	1 (1,2)	0 (0)	
**30-day re-admission**[N(%)]	3 (4)	5 (6)	0.71
**30-day mortality**[N(%)]	0 (0)	0 (0)	0.99
**Destination at discharge** [N(%)]			0.28
Home	69 (86)	61 (76)	
Long-term care institutions	8 (10)	14 (17)	
Other hospital ward	3 (4)	5 (6)	
**TNM cancer stage**			0.37
In situ	2 (2,5)	2 (2,5)	
I	16 (20)	15 (18,7)	
IIa	22 (32,3)	27 (39,1)	
IIb	4 (5)	5 (6)	
IIc	0 (0)	0 (0)	
IIIa	3 (4)	1 (1,2)	
IIIb	18 (22,5)	12 (15)	
IIIc	2 (2,5)	8 (10)	

NGT: nasogastric tube; RBC: red blood cells; ICU: intensive care unit.

The unadjusted and adjusted analyses are illustrated in Table 5, Table 6. Adjusted logistic regression analysis showed that BMI ≥30 and the conventional peri-operative protocol were associated to increased risk of post-operative complications, while following a conventional peri-operative care protocol was the only factor associated to a prolonged hospital LoS (p < 0.0001).

**Table 5:** Association between baseline characteristics, intraoperative variables, and type of perioperative protocol and any postoperative complications according to logistic regression analysis adjusted for potential confounders (surgical approach, intraoperative fluid infusions and duration of surgery).

Any postoperative complication				
Variable	Unadjusted Model Full Adjusted		Model	
	OR (95 % CI)	*P*	OR (95 % CI)	*P*
**Gender** (ref. female)				
male	1.11 (0.59–2.11)	0.741	1.31 (0.58–2.94)	0.51
**Age** (ref. < 75 y)				
≥75	1.97 (1.03–3.76)	0.041	1.76 (0.78–3.99)	0.17
BMI* (ref. < 25 kg/m^2^)				
25–29.9	0.83 (0.41–1.68)	0.602	0.92 (0.39–2.17)	0.84
≥30	5.21 (1.39–19.53)	0.014	5.40 (1.12–25.98)	0.03
**ASA**^a^**(**ref. I)				
II	0.34 (0.03–3.93)	0.388	0.32 (0.03–4.06)	0.37
III	1.43 (0.12–16.58)	0.776	1.54 (0.11–20.79)	0.74
**Intraoperative intravenous fluids** (ml/kg/h) (ref. ≤ 10 ml/kg/h)				
10.1–15.0	0.61 (0.26–1.45)	0.261	0.399 (0.13–1.23)	0.10
≥15.1	1.22 (0.52–2.84)	0.649	0.88 (0.25–3.03)	0.83
**Perioperative protocol** (ref. ERP)				
Tradit ional	2.44 (1.27–4.66)	0.007	3.02 (1.22–7.52)	0.01

* BMI - Body Mass Index.

a ASA - American Society of Anesthesia.

**Table 6:** Association between baseline characteristics, intraoperative variables, and type of perioperative protocol and prolonged length of hospital stay according to Cox regression analysis adjusted for potential confounders (surgical approach, intraoperative fluid infusions and duration of surgery).

Prolonged hospital length of stay				
Variable	Unadjusted Model		Full Adjusted Model	
	HR (95 % CI)	*P*	HR (95 % CI)	*P*
**Gend Gender** (ref. female)				
Male male	0.83 (0.60–1.13)	0.235	0.81 (0.58–1.15)	0.24
**Age** (**Age** (Ref: <75 ys)				
≥75	0.74 (0.54–1.02)	0.067	0.88 (0.61–1.27)	0.48
BMI* **BMI** (ref: <25 kg/m^2^)				
25-2 25–29,9	1.42 (0.99–2.04)	0.054	1.54 (1.05–2.25)	0.02
30 ≥ 30	0.97 (0.59–1.57)	0.887	1.11 (0.67–1.83)	0.69
**ASA**^a^**ASA score** (ref: I)				
III II I	0.47 (0.15–1.52)	0.210	0.44 (0.13–1.45)	0.17
IIIIII III	0.39 (0.12–1.26)	0.115	0.30 (0.09–1.00)	0.05
**Perio Perioperative protocol** (ref. ERP)				
TraditiTraditional	0.32 (0.23–0.45)	<0.0001	0.28 (0.20–0.40)	<0.0001

*BMI - Body Mass Index.

a ASA - American Society of Anaesthesiologists.

## 4 Discussion

In the current study we evaluated the clinical outcome in patients who underwent elective colorectal resection in a single institution serving a wide low densely populated rural and fishing area (i.e., South-Eastern Province of Ferrara) *before*, retrospectively, and *after*, prospectively, the adoption of a colorectal ERP. The population density of such a geographical area is about 77.4 inhabitants/km^2^, which is comparable to that of the Tuscan-Emilian Apennines, despite hosting the Po river valley and delta. These characteristics, together with the social profile and demographics of the population (101,458 inhabitants), constituted an ideal study context.

Implementing the multimodal protocol in such an area, improved safely patient's convalescence by reducing time to functional recovery, lowering by half the duration of hospital LoS, and decreasing postoperative medical complications, with no increase in mortality and 30-day re-admissions. Finally, following a traditional perioperative care protocol was the only factor we found to be associated to a prolonged postoperative hospital LoS.

The application of an ERP may be particularly difficult in rural hospitals serving wide areas, as it may be hindered by multiple factors affecting both health care professionals and patients such as: 1) lack of strong scientific evidence supporting the real efficacy outside urban areas and tertiary or academic hospitals; 2) fear of complications due to decrease resources to manage postoperative complications; 3) more difficult access to medical care by the patients; 4) decreased health literacy as ERP principles may not be intuitive; 5) higher medical and nursing staff turnover and shortage; 5) poor familiarity with some elements of ERP protocol by medical and nursing staffs; 6) lack of time and commitment by health care professionals to constitute a multidisciplinary team; 7) limited hospital resources; 8) lower case-volume.

Introduction of ERP into clinical practice has been pioneered as *fast-track* surgery by Henrik Kehlet and colleagues in the mid-1990s,^1^ with the principal objective to optimize postoperative outcomes of the surgical patients. This protocol was initially used in urban and academic tertiary care centers and many hospitals began to adopt it, with a slow progressive dissemination from Northern Europe and North America throughout the world. The core guidelines established by Kehlet were delineated by consensus review [13], until the birth of the Enhanced Recovery After Surgery (ERAS) society in 2010 [14]. The safety and efficacy of colorectal ERP has been established in a few randomized studies and meta-analysis of randomized studies conducted in urban and academic hospitals [[15], [16], [17]]. Until today, however, the evidence regarding the adoption and feasibility of such a program in rural contexts is quite limited [[3], [4], [5], [6], [7], [8]], which may be perceived as a barrier to ERP implementation in those area. Very few experiences from North American rural and community hospitals ^6-8^ as well as European rural contexts [[3], [4], [5]] have been published in the last decade. Tebala GD et al. [3] found age and laparoscopic approach as independent prognostic factors significantly associated with early discharge with a readmission rate of 9.1 %. Marres CCM et al. [[3], [4], [5], [6], [7], [8]] also found a significant reduction of major post-operative complications and mortality after implementing a quality improvement program in colorectal surgery. Geltzeiler CB et al. [6] analyzed the evolution of implementing colorectal ERP from 2009 to 2012 and they found a significant decrease of hospital LoS (6.7 days vs 3.7 days) with a remarkable estimated cost-saving for patients. Archibald LH et al. [7] investigated the introduction of a comprehensive care process for enhanced recovery after colon surgery in eight community hospitals and they concluded that ERP represents the most important factor, more than laparoscopic approach, in decreasing length of stay.

As evidenced from the literature, there is a strong relationship between the adherence to the elements of the protocol and the complete recovery of patients with a remarkable reduction in hospital LoS [18,19]. The median adherence to ERP protocol in our study was 68 %. Here are some of the difficulties often encountered during the implementation of ERAS protocols, especially in such rural contexts: resistance to change, cultural and organizational barriers, multidisciplinary coordination, resource allocation, standardization, patient education and monitoring. We successfully implemented the ERP protocol by constituting a motivated multidisciplinary care team (i.e., nurses, surgeons, anesthesiologists, and dietician), through scheduled periodic audits for improving staff education and to assess the correct implementation of the protocol and to maintain high compliance. Moreover, the institution of a “case manager” was of paramount importance for the multidisciplinary coordination and patient education. Two important items were not fulfilled: the amount of intra-operative fluids administration and early mobilization after surgery. Concerning the first element, although the amount of intra-operative fluids was reduced with the adoption of the program versus control, the target infusion was not reached, which was probably related to the habits of anesthetists. Early mobilization was probably affected by advanced patients’ age [77 years-old (69–83)] as well as the high nurse to patient ratio (1:12 a.m., 1:12 p.m.) and limited physiotherapists available for support.

Another remarkable achievement with the ERP was patient hospital discharge as soon as recovery was complete according to predefined standardized criteria (i.e., fit for discharge), while control patients left the hospital a median of one day after they were fit for discharge.

It could be argued that the improved outcome among ERP patients could be due to the use of laparoscopy (95 %) as opposed to open surgery among control patients. Certainly, the laparoscopic approach is a key stress reducing element that should be integrated in ERP to obtain the greatest improvement in recovery.^17^ In 2015, a systematic review and meta-analysis of randomized trials comparing laparoscopic versus open colorectal surgery within ERAS programs showed that the mean difference in hospital stay between the two groups was only below two days in favor of laparoscopy.[20] The Authors concluded that the benefits of laparoscopic colorectal resection remain to be proved within optimal ERAS programs In fact, when comparing colorectal laparoscopic versus open surgery within ERAS programs the differences may well cancel each other out [[21], [22], [23]].

The global peri-operative patient care, however, is fundamental to improve the postoperative outcome regardless of the approach used [24,25]. A meta-analysis of randomized trials on open colorectal resections showed a significant reduction of hospital LoS by following ERP [24]. Finally, it should be noticed that being on traditional rather than enhanced recovery care was the only factor associated to prolonged hospital LoS in our study population.

Another important aspect of ERP perioperative care is related to health cost-saving. Previous studies show hospital LoS reduction yielding significant cost savings per patient with ERP in colorectal surgery [6,8,10]. Moreover, a prospective study underlined the benefits of an ERP in a North American community hospital in terms of overall wound complications rates [25]. Although not evaluated in our investigation, the decrease in duration of hospital LoS among patients on enhanced recovery may well suggest a reduction for institutional costs with the ERP.

### 4.1 Limitations and strength

This is a single center prospective study with a historical control group used for comparison and, therefore, the results must be interpreted with caution.

Reviewing medical records to collect data on functional recovery and pain control (i.e., readiness for discharge) for the Pre-ERP group as opposed to prospective data collection in the ERP-group may have affected the quality of data collection.

Due to profound organizational changes in the unit and the time of implementation of the program there is a 24-months interval between the study periods. Also, two different surgeons operated in the ERP group and pre-ERP group, respectively.

Patients in the ERP group may have benefited from the laparoscopic approach as opposed to the open one adopted in the Pre-ERP group. Minimally invasive approaches, however, are an important component of ERPs to reduce the postoperative surgical stress response.

Nonetheless, given the weakness and paucity of scientific evidence about implementation of ERP in colorectal surgery, this study is very useful as it clearly shows the reproducibility of a safe and effective colorectal ERP within a wide agricultural area with a low-density population.

## 5 Conclusions

Although limited resources are perceived as a barrier to ERP implementation, the current experience showed how the use of an ERP in a hospital serving a wide rural low densely populated area is feasible and effective, despite it requires greater effort.

## What does this paper add to the literature?

- Currently, the scientific evidence regarding the adoption and feasibility of such a program in rural contexts is quite limited, which may be perceived as a barrier to enhanced recovery program (ERP) implementation in those area.
- Our experience demonstrated how the use of ERP in colorectal surgery in a hospital serving a wide rural low densely populated area is feasible, safe and effective, despite it requires greater effort.

## CRediT authorship contribution statement

**Antonio Pesce:** Conceptualization, Methodology, Writing – original draft. **Mattia Portinari:** Formal analysis, Methodology, Validation. **Nicolò Fabbri:** Data curation, Investigation, Methodology. **Valeria Sciascia:** Data curation, Investigation. **Lisa Uccellatori:** Data curation. **Michela Vozza:** Data curation, Investigation. **Erminio Righini:** Investigation, Supervision, Validation. **Carlo V. Feo:** Conceptualization, Supervision, Writing – review & editing.

## Declaration of competing interest

The authors declare that they have no known competing financial interests or personal relationships that could have appeared to influence the work reported in this paper.

## References

[1] Kehlet H. (1997). Multimodal approach to control postoperative pathophysiology and rehabilitation. Br. J. Anaesth..

[2] Kehlet H., Wilmore D.W. (2008). Evidence-based surgical care and the evolution of fast-track surgery. Ann. Surg..

[3] Tebala G.D., Keane S., Osman A. (2016). Early discharge after colorectal resection: the positive impact of an enhanced recovery program on a rural colorectal surgery service. Surg Laparosc Endosc Percutan Tech.

[4] Marres C.C., van de Ven A.W., Verbeek P.C. (2016). The effect of a postoperative quality improvement program on outcomes in colorectal surgery in a community hospital. Int J Color Dis.

[5] Frontera D., Arena L., Corsale I. (2014). Fast track in Colo-rectal surgery. Preliminary experience in a rural hospital. G Chir.

[6] Geltzeiler C.B., Rotramel A., Wilson C. (2014). Prospective study of colorectal enhanced recovery after surgery in a community hospital. JAMA Surg.

[7] Archibald L.H., Ott M.J., Gale C.M. (2011). Enhanced recovery after colon surgery in a community hospital system. Dis. Colon Rectum.

[8] Smucker L., Victory J., Scribani M. (2020 Dec 3). Rural context, single institution prospective outcomes after enhanced recovery colorectal surgery protocol implementation. BMC Health Serv. Res..

[9] Feo C.V., Portinari M., Tsolaki E. (2016). The effect of an Enhanced Recovery Program in elective retroperitoneal abdominal aortic aneurysm repair. J. Vasc. Surg..

[10] Portinari M., Ascanelli S., Targa S. (2018). Impact of a colorectal enhanced recovery program implementation on clinical outcomes and institutional costs: a prospective cohort study with retrospective control. Int. J. Surg..

[11] Targa S., Portinari M., Ascanelli S. (2021 Apr). Enhanced recovery program in laparoscopic colorectal surgery: an observational controlled trial. J. Laparoendosc. Adv. Surg. Tech..

[12] Dindo D., Demartines N., Clavien P.A. (2004). Classification of surgical complications: a new proposal with evaluation in a cohort of 6336 patients and results of a survey. Ann. Surg..

[13] Lassen K., Soop M., Nygren J., Enhanced Recovery After Surgery (ERAS) Group (2009). Consensus review of optimal perioperative care in colorectal surgery: enhanced Recovery after Surgery (ERAS) Group recommendations. Arch. Surg..

[14] Nygren J., Thacker J., Carli F., Enhanced Recovery After Surgery (ERAS) Society for Perioperative Care; European Society for Clinical Nutrition and Metabolism (ESPEN); International Association for Surgical Metabolism and Nutrition (IASMEN) (2013). Guidelines for perioperative care in elective rectal/pelvic surgery: enhanced Recovery after Surgery (ERAS®) Society recommendations. World J. Surg..

[15] Greco M., Capretti G., Beretta L. (2014). Enhanced recovery program in colorectal surgery: a meta-analysis of randomized controlled trials. World J. Surg..

[16] Varadhan K.K., Neal K.R., Dejong C.H.C. (2010). The enhanced recovery after surgery (ERAS) pathway for patients undergoing major elective open colorectal surgery: a meta-analysis of randomized controlled trials. ClinNutr.

[17] Wind J., Hofland J., Preckel B. (2006 Nov 29). Perioperative strategy in colonic surgery;LAparoscopy and/or FAst track multimodal management versus standard care (LAFA trial). BMC Surg..

[18] Pecorelli N., Hershorn O., Baldini G. (2017). Impact of adherence to care pathway interventions on recovery following bowel resection within an established enhanced recovery program. Surg. Endosc..

[19] Fiore J.F.J., Faragher I.G., Bialocerkowski A. (2013). Time to readiness for discharge is a valid and reliable measure of short-term recovery after colorectal surgery. World J. Surg..

[20] Zhuang C.L., Huang D.D., Chen F.F. (2015 Aug). Laparoscopic versus open colorectal surgery within enhanced recovery after surgery programs: a systematic review and meta-analysis of randomized controlled trials. Surg. Endosc..

[21] Kennedy R.H., Francis E.A., Wharton R. (2014). Multicenter randomized controlled trial of conventional versus laparoscopic surgery for colorectal cancer within an enhanced recovery programme: EnROL. J. Clin. Oncol..

[22] Guillou P.J., Quirke P., Thorpe H. (2005). Short-term endpoints of conventional versus laparoscopic-assisted surgery in patients with colorectal cancer (MRC CLASICC trial): multicentre, randomised controlled trial. Lancet.

[23] van der Pas M.H., Haglind E., Cuesta M.A. (2013). Group COcLoORIS. Laparoscopic versus open surgery for rectal cancer (COLOR II): short-term outcomes of a randomised, phase 3 trial. Lancet Oncol..

[24] Faiz O., Warusavitarne J., Bottle A. (2009). Laparoscopically assisted vs. open elective colonic and rectal resection: a comparison of outcomes in English National Health Service Trusts between 1996 and 2006. Dis. Colon Rectum.

[25] D'Souza K., Choi J.I., Wootton J. (2019 Feb 1). Impact of sequential implementation of multimodal perioperative care pathways on colorectal surgical outcomes. Can. J. Surg..

